# The importance of audio-video documentation during polysomnography for diagnosis of catathrenia in a 6-year-old child: case report

**DOI:** 10.5935/1984-0063.20200122

**Published:** 2021

**Authors:** Nubia Santana Argollo, Diderot Rodrigues Parreira, Lucas Carneiro Pereira

**Affiliations:** 1 Instituto de Otorrino de Brasilia, Otorhinolaringology - Brasília - Distrito Federal - Brazil.; 2 Universidade Católica de Brasilia, Otolarhinolaryngology - Brasília -Distrito Federal - Brazil.; 3 Universidade Católica de Brasilia, Otolarhinolaryngology - Brasília - Distrito Federal – Brazil.

**Keywords:** Polysomnography, Sleep Apnea Syndromes, Central sleep apnea

## Abstract

Catathrenia is a rare disorder classified as a respiratory one and characterized by expiratory groans during sleep. We report a case of catathrenia in a 6-year-old male patient, with documented video and audio polysomnography. The diagnosis of catathrenia is made through a detailed analysis of video and audio recordings during the examination. Although there is no association with the risk of physical damage known today, catathrenia presents itself as a source of anxiety among patients and their families. The differentiation of catathrenia with central apnea, obstructive apnea, primary snoring, and parasomnias will influence both the prognosis and the diagnosis and treatment of the patient.

## INTRODUCTION

Catathrenia is a rare condition, classified as a sleep-related respiratory disorder^1^. It is characterized by expiratory groans during sleep^1, 2^. As it is a rare diagnosis, it is often confused with stridor, sleep-related laryngospasm, and sleepiness^3, 4^. Current literature presents few cases reported in adults, and the onset of the disorder is commonly described in young adults. It is even more infrequent to have reported cases in children^5, 6^. We hereby report a case of catathrenia in a six-year-old male patient with video and audio documented polysomnography.

## CASE REPORT

A patient, named V.S.G, male, 3-years-old, residing in a social assistance institution since 2 years of age, with no previous data. Complaint of high intensity snoring and night agitation since the time of arrival at the institution. An otorhinolaryngological physical examination was performed with findings suggestive of allergic rhinopathy. There were no noticeable alteration on the nasal videoendoscopy. Treatment for allergic rhinitis was instituted, using nasal corticosteroids, oral antihistamines, and oral antileukotriene for three months. The patient followed-up, reporting a significant decrease in snoring and night agitation.

Two years later, as a newly adopted child, his parents with the same prior complaint brought him. The patient was reevaluated and no changes were observed when compared to previous examinations. Similarly, he underwent treatment for allergic rhinitis. After 2 months, there was a significant decrease in snoring and night agitation, however, he persisted with groaning episodes, despite the decrease in frequency when compared to the condition before to treatment. The father performed video and sound monitoring at night with a home camera and sent it to us. When evaluating the video, we observed that the child had several outbreak episodes at night apparently of expiratory groaning and, during these events he had rhythmic movement of the hip several times, before or after the outbreaks of groaning.

A polysomnography exam with extended electroencephalogram assembly and observation of sound and video was requested. The examination was performed, however, did not comply with the explicit requests in the medical order. There was no mention of the groans in the report and a slightly increased apnea/hypopnea index (IAH-1.8) was observed, without significant desaturation.

During the follow-up, we observed that the child did not have complete remission of the groans, but if he had an upper airway infection or allergic rhinitis crisis, the snores and groans increased in frequency and intensity.

**Figure 1:**
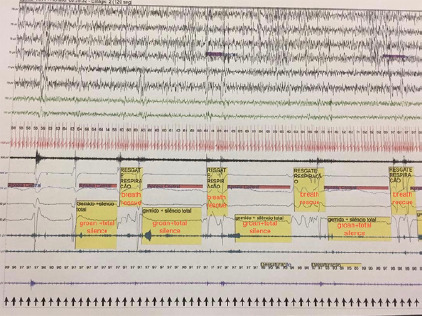
First catathrenia outbreak of night-polysomnographic pattern.

**Figure 2:**
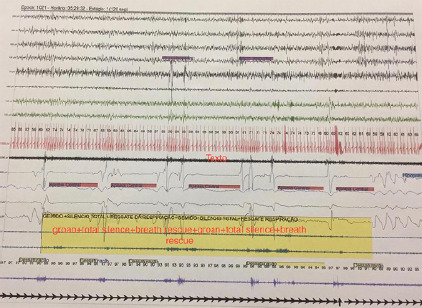
Second Catathrenia outbreak of night-polysomnographic pattern.

Approximately a year and a half after the first polysomnography, the child was already 6-years-old, and he maintained adequate neuropsychomotor development, had adequate social and affective behavior, and a good control of the allergic rhinitis. Another polysomnography was requested. This time, simultaneous sound and video monitoring were performed for all parameters analyzed in a complete polysomnography (6 EEG channels, 3 ECG channels, 2 EOG channels, 2 masseter electrodes, snoring sensor, nasal cannula, oronasal airflow sensor,

EMG sensor in one of the lower limbs). We observed that the child had 3 outbreaks of expiratory groans, all of them occurring in the second half of the nighttime sleep. The first outbreak started at 4.37 a.m. during the NREM sleep and presented an expiratory groan pattern, which lasted an average of 10 seconds followed by complete silence (Figure 1). The flow sensor and nasal cannula tracing in the polysomnography was compatible with central apnea (summation of groan and silence). The silence period usually lasted an average of 20 seconds, followed by a short period of inspiration, and was again followed by an episode of moaning with complete silence. This outbreak had a duration 4 minutes and 15 seconds. Afterwards, sleep remained still and no events were recorded for several minutes. At 5.26 a.m., there was another outbreak, also in NREM sleep, and it showed the same cyclic pattern as the previous one (Figure 2). This second outbreak lasted 4 minutes and 5 seconds. The final outbreak of the night started at 5.57 a.m. and lasted only 1 minute and 5 seconds, also during NREM sleep. This outbreak was followed by complete arousal, which lasted 1 minute. Then, the child began to make rhythmic movements with the hip together with the lower limbs. After 2 minutes, there was another episode of groaning/total silence, which lasted for 15 seconds.

It is necessary to emphasize that during the night the patient also presented episodes of hypopnea/obstructive apnea with mild inspiratory snoring in some moments, totaling an obstructive apnea/hypopnea index of 4.5 events per hour (slight increase for age group 6, without significant desaturation). There were no electroencephalographic changes suggestive of an epileptic seizure, nor cardiac alterations at any time during the night (respecting the method limitation).

Subsequently, the patient underwent cardiac evaluation with a specialist in the field. The cardiological evaluation with physical examination, electrocardiogram, echocardiogram, and chest radiography did not reveal any noteworthy changes in their findings.

## DISCUSSION

Catathrenia is considered a rare sleep-related respiratory disorder, according to the third edition of the International Classification of Sleep Disorders^1^. In its previous classification, catathrenia was considered a type of parasomnia, a debated and controversial fact in current literature^2^. It was described for the first time as a single case report by Roeck et al., in 1983^7^. After such publication, there were few cases reported worldwide in adults The description of cases in the pediatric population is even more infrequent^6, 8^.

In 2017, Langley et al.^6^ described the first and only case report, in a 5-year-old child, with catathrenia observed by the parents since birth. This case is similar in many ways to the case described by our group.

In 2009, due to a series of cases reported by Jaar et al.^9^, the incidence of catathrenia was identified in 25 out of 15,052 (0.17%) patients with sleep disorders and/or awakening disorders, in a center specialized in sleep medicine, during a 10 year period in Japan. In 2012, Øverland et al.^10^ conducted a study which showed an incidence of catathrenia in 4 cases out of 1,004 patients (0.4%), who underwent polysomnography over a period of 1 year in an institution in Norway.

Catathrenia diagnosis must be made through a detailed analysis of the video and audio recordings during the examination conducted by trained professionals, due to its similarity with the polysomnographic pattern of central sleep apnea^8, 11, 12^.

It is classically described through polysomnography as a deep inhalation followed by a prolonged exhalation with groans, lasting between 2 and 49 seconds^13^. Catathrenia occurs predominantly during REM sleep^14^, however, it was also identified in non-REM sleep^5^. In a systematic review by Oldani et al., in 2012^15^, it is reported that the frequency of catathrenia episodes found in the study was 3.6 times per night/5.9 days per week.

The differential diagnosis is made with somniloquy (parasomnia which produces loud vocalization during sleep) and snoring (noise produced due to the vibration of the soft tissues of the upper airway)^3^. It is important to highlight that catathrenia is not just an expiratory snore. Studies based on acoustic analysis show that the sounds are different. Catathrenia is a laryngeal sound, while snoring is guttural^3, 15^.

There are treatment reports with the continuous positive airway pressure machine (CPAP) during sleep, which have had limited success. There is also reports of the use of behavioral therapy, in addition to various medications such as benzodiazepines, antidepressants, and antiepileptics with all of which have presented very limited and variable results^3, 16^.

A study by Ott et al. (2011)^3^ demonstrated reductions in nocturnal groans, as well as improved daytime well-being after CPAP use, according to the authors, possibly due to a reduction in active adduction of vocal folds during expiration^3, 16^.

Pevernagie et al. (2001)^17^ and Pevernagie (2017)^18^ report that complaints such as insomnia or daytime sleepiness vary from minimal to severe. In another study, Drakatos et al. (2016)^14^ describe groaning (52%), snoring (18%), and daytime sleepiness (45%) as the main complaints related to the disorder. In this same study, it was found that there is generally no association with abnormal motor behavior, speaking during sleep or description of vivid dreams, but snoring may occur^14^. On the other hand, in the case described by our group, rhythmic body movement was observed after and/or at the beginning of catathrenia episodes, which are similar to disturbances of the wake-sleep transition (such as hypnagogic myoclonus or rhythmic sleep movements), but without greater clinical significance. And also mild inspirational snoring at times during the night.

Clinical and neurological findings are normal or nonspecific. There is no clear association with any predisposing factors or underlying disease, and it does not cause long-term morbidity^2, 16^.

Catathrenia is a source of anxiety among patients and their families. Polysomnography can be useful if performed properly to confirm the diagnosis and evaluate comorbid sleep disorders, such as obstructive sleep apnea and parasomnia, among other ones. Further studies may involve performing deep breathing exercises, yoga, meditation or myofunctional therapy to decrease symptoms. In addition, bed partners may consider the use of earplugs^16^.

## References

[1] Sateia MJ (2014). International classification of sleep disorders – third edition:
highlights and modifications. Chest.

[2] Dias C, Sousa L, Batata L, Teixeira MF, Santos JM (2015). Catathrenia: a 10 year revision. Eur Respir J [Internet].

[3] Ott SR, Hamacher J, Seifert E (2011). Bringing light to the sirens of night: laryngoscopy in
catathrenia during sleep. Eur Respir J [Internet].

[4] Vetrugno R, Provini F, Plazzi G, Vignatelli L, Lugaresi E, Montagna P (2001). Catathrenia (nocturnal groaning): a new type of
parasomnia. Neurology [Internet].

[5] Manis E, Chervin RD (2018). 1149 a female with neurodevelopmental disorders, obstructive
sleep apnea and supine NREM catathrenia. Sleep.

[6] Langley RJ, Hill L, Hill EA, Urquhart DS (2017). The curious incident of groaning in the night-
time. Breathe (Sheffield, Engl) [Internet].

[7] Roeck J, Van Hoof E, Cluydts R (1983). Sleep-related expiratory groaning: a case report. Sleep Res.

[8] Salhan D, Relia S, Dayyat E, Schoumacher R, Freire AX (2018). 1135 teenager with a noisy breathing in sleep - a rare case of
Catathrenia. Sleep.

[9] Jaar O, Pilon M, Montplaisir J, Zadra A (2009). What is nocturnal groaning (catathrenia)? -analysis of PSG
data. Sleep.

[10] Øverland B, Akre H, Berdal H, Skatvedt O (2012). Sleep-related groaning: prevalence and characteristics in a
cohort of patients with suspected obstructive sleep apnea. Acta Otolaryngol [Internet].

[11] Okura M, Muraki H (2013). WS1-3. Attended video-audio polysomnographic study about patients
with catathrenia (sleep related groaning). Clin Neurophysiol.

[12] Muraki H, Okura M, Kato T, Taniguchi M, Ohi M (2017). A stereotyped sequence from EEG arousals to nocturnal groaning
events with or without the intervening sleep bruxism in
catathrenia. Sleep Med.

[13] Iriarte J, Fernández S, Fernandez-Arrechea N, Urrestarazu E, Pagola I, Alegre M (2011). Sound analysis of catathrenia: a vocal expiratory
sound. Sleep Breath [Internet].

[14] Drakatos P, Higgins S, Duncan I, Stevens S, Dastagir S, Muza R (2016). Catathrenia as a REM predominant disorder of
arousal. Eur Respir J [Internet].

[15] Oldani A, Manconi M, Zucconi M, Castronovo V, Ferini-Strambi L (2005). Nocturnal groaning: just a sound or parasomnia?. J Sleep Res.

[16] Alonso J, Camacho M, Chhetri DK, Guilleminault C, Zaghi S (2017). Catathrenia (nocturnal groaning): a social media survey and
state-of-the-art review. J Clin Sleep Med [Internet].

[17] Pevernagie DA, Boon PA, Mariman AN, Verhaeghen DB, Pauwels RA (2001). Vocalization during episodes of prolonged expiration: a
parasomnia related to REM sleep. Sleep Med.

[18] Pevernagie DA (2017). Why catathrenia is a parasomnia. Sleep Med.

